# Emerging roles of telemedicine in dementia treatment and care

**DOI:** 10.1590/1980-5764-DN-2022-0066

**Published:** 2023-05-29

**Authors:** Abdulbasit Opeyemi Muili, Mubarak Jolayemi Mustapha, Michael Chukwubuikem Offor, Habeebulah Jayeola Oladipo

**Affiliations:** 1 Ladoke Akintola University of Technology, Department of Medicine, Ogbomosho, Nigeria.; 2 University of Ilorin, Faculty of Basic Medical Sciences, Ilorin, Nigeria.; 3 University of Ibadan, College of Medicine, Ibadan, Nigeria.; 4 Faculty of Pharmaceutical Sciences, University of Ilorin, Ilorin, Nigeria.

**Keywords:** Dementia, Telecommunications, Diagnosis, Telemedicine, Health Care Sector, Demência, Telecomunicações, Diagnóstico, Telemedicina, Setor de Assistência à Saúde

## Abstract

Dementia is a neurological disorder that affects memory, thinking, orientation, and other important functions of the brain; telemedicine is a part of the healthcare delivery system involving diagnosis and consultation over telecommunications devices such as mobile phones and computers. In this review, we assessed the impact, accessibility, and possible improvements in telemedicine in dementia treatment. Regarding the use of telemedicine in the treatment, we evaluated its impact on the management of the disease (i.e., diagnosis and follow-up). We also evaluated studies on the current improvements and accessibility of telemedicine in dementia treatment. The review findings showed that it is effective in diagnosing patients, monitoring their progress during treatment, and providing caregiver support. However, studies have revealed a lack of accessibility and improvement in telemedicine among the elderly, particularly in West African countries. Finally, lasting solutions were provided to address the problems in the review permanently.

## RESUMO.

A demência é um distúrbio neurológico que afeta a memória, o pensamento, a orientação e outras funções importantes do cérebro, enquanto a telemedicina faz parte do sistema de prestação de cuidados de saúde que envolve diagnóstico e consulta por meio de dispositivos de telecomunicações, como telefones celulares e computadores. Nesta revisão, avaliamos o impacto, a acessibilidade e as possíveis melhorias da telemedicina no tratamento da demência. Ao avaliar o impacto da telemedicina no tratamento da demência, avaliamos também seu impacto no manejo da demência (ou seja, diagnóstico e acompanhamento do tratamento da demência). Também avaliamos estudos sobre as melhorias atuais a acessibilidade da telemedicina no tratamento da demência. Os resultados da revisão mostraram que a telemedicina é eficaz para diagnosticar pacientes, monitorar seu progresso durante o tratamento e fornecer suporte ao cuidador. No entanto, estudos revelaram falta de acessibilidade e melhoria da telemedicina entre os idosos, principalmente nos países da África Ocidental. Finalmente, para resolver o problema, foram fornecidas soluções duradouras para resolver permanentemente os problemas na revisão.

## INTRODUCTION

According to the World Health Organization (WHO), there are about 55 million dementia patients worldwide, with approximately 10 million new cases reported yearly, and the number of cases is expected to reach 139 million by 2050^1^. Currently, there are no disease-modifying treatments for dementia^2^. In response to this, new and credible methods of treatment are being introduced to care for dementia patients. The term “dementia” is used to refer to any disorder in which a person's cognitive abilities significantly deteriorate and interfere with their ability to do daily tasks, care for themselves, or interact with others^2^. It is also a collective term for a number of illnesses and diseases of the brain that have an impact on the cognition, balance, motion, reasoning, learning, function, and decision-making abilities^3^. The various symptoms of dementia depend on the area of the brain affected, the main being the frontal, temporal, and parietal lobes^4^. Changes in behavior are usually noticed when the frontal lobe of the brain is affected, while changes in language, memory, visuospatial, and visuoperceptive functions are noticed when the parietal and temporal lobes are affected. Dementia is a clinical syndrome with many etiologies. These etiologies can be classified into neurodegenerative and non-neurodegenerative diseases^5^. Neurodegenerative dementia includes Alzheimer's disease, vascular dementia, Lewy body disease, and frontotemporal dementia. Non-neurodegenerative dementias include infections and immune disorders, nutritional deficiencies, subdural hematomas, and medication side effects^4^.

Telemedicine, also known as telehealth or electronic health (e-health), is a healthcare delivery system involving diagnosis and consultation over telecommunications devices such as mobile phones and computer devices. It is also known as the solution that helps improve healthcare accessibility by reducing pain points experienced by patients and caregivers when participating in dementia and other diseases, and infection care^6^. Telemedicine has also made significant contributions to achieving universal health coverage (UHC) in several countries around the world by improving access to quality and affordable health services for a wide range of patients in most countries^7^.

The number of individuals needing health services increases along with the global population. Therefore, getting a proper diagnosis and treatment in a health care center can be a tedious task. Telemedicine as an avenue for health delivery helps to bridge the gap between the patient and the health practitioner. The implementation of telemedicine in healthcare had been efficient during the COVID-19 pandemic, which has reduced congestion in hospitals and clinics and has provided continuous care for patients. There is also the possibility of accessing optimum care through the service of telemedicine, which includes primary care consultations, psychotherapy, physical therapy, and emergency services^8^.

This paper aims to assess the role telemedicine plays in the treatment of dementia, its accessibility to patients, and its improvements in relation to the disease care.

## METHODS

A standard and comprehensive literature search was conducted on three databases: PubMed, Google Scholar, and Scopus. In this review we intended to access the emerging roles of telemedicine in the treatment and care of dementia by critically appraising various articles that evaluated the impact of telemedicine on the diagnosis and follow-up of the disease, challenges facing the usage of telemedicine in treatment care and the accessibility of telemedicine to patients.

In the literature search we made use of keywords combined with Boolean operators (AND, OR). The following key words were used in the search: (“telemedicine” OR “telehealth,” OR “e-health”) AND (“Dementia” OR “ADRD”) AND (“treatment” OR “management” OR “diagnosis” OR “challenges” OR “follow-up”). These combinations were used to capture the roles of telemedicine in dementia treatment and their impact on the management of the disorder – diagnosis, follow-up, and challenges were among the searched terms. Additionally, the MeSH terms were used during the literature search on PubMed to ensure a consistent and accurate result.

### Eligibility criteria

The articles that were included in this review were:

- Studies conducted using various telecommunications devices to assess the impact of telemedicine on the diagnosis of dementia;
- Clinical studies conducted on the roles of telemedicine in the follow-up of dementia;
- Studies on the implementation of telemedicine in the general management of dementia;
- Studies that identified challenges in the implementation of telemedicine in dementia treatment; and
- Studies that identified the accessibility of telemedicine among various dementia patients. Papers not written in English were excluded.

There was no restriction on the length of the study. The search included a thorough screening of clinical studies, systematic reviews, and full-text publications. Those that were highly relevant to the studies were chosen while those that didn't meet the eligibility criteria were excluded. Additional screening was also done on the reference list of selected articles to ensure an accurate selection.

## IMPACT OF TELEMEDICINE ON THE MANAGEMENT OF DEMENTIA

The use of telemedicine and e-health is now popular among the middle-aged and the elderly; more than “50% of those aged over 65 years” use telecommunication and e-health services for seeking medical treatment and support^9^. From the previous section, we can infer that there has been a tremendous increase in the number of people living with dementia, which shows that in-person consultation with health professionals may be very strenuous. The delivery of health services using telecommunication devices has been known to provide services in an easy and faster way.

Telemedicine has found its use in diagnosis, follow-up and support for dementia patients through caregiving. The diagnosis of dementia involves noting down the medical history of the patients through speaking with them; most importantly, obtaining a confirmatory report from their families and friends, determining their functionality, conducting a cognitive examination, and appraising relevant information^10^. Diagnosis can be performed via video teleconferencing (VTC) or by phone. VTC involves virtual face-to-face contact with a health practitioner and has shown significant promise in replacing face-to-face (FTF) dementia diagnosis^11^. When the patients start developing noticeable symptoms of dementia and the issue is reported to the appropriate health professionals by their families or friends, the initial non-specialist assessment will take place. It typically involves history taking, a Mini-Mental State Examination (MMSE), the revised Hopkins verbal learning test (HVLT-R), letter fluency (LF), and a cognitive screening test^12^. This assessment will attempt, among other things, to confirm the presence of an objective cognitive problem and rule out reversible causes of cognitive decline, as well as determine and record the type and stage of dementia present in the patient^12^. Comparative studies^13,14^ discovered agreement between FTF and VTC assessments. The tests used in their research included the MMSE, the clock drawing test, the Digit Span (DGS) Forward and Backwards, verbal or letter fluency, and category fluency^15–17^. The reliability across the FTF and VTC was determined by examining the results for the intraclass correlation coefficient (ICC), which is a measure of reliability agreement; the Bradley-Blackwood procedure^18^ and the statistical review was done using the SPSS packages. From the results, it was inferred that the VTC is similar to the FTF in many respects and that it is reliable for teleneuropsychological assessment in diagnosing dementia and other related conditions; however there is a need for further research on testing the reliability of complex tasks requiring motor and visual ability^19^.

The progressive nature of Alzheimer's disease and related dementias (ADRD) necessitates recurrent follow-up and proper management. During the ADRD follow-up, we identified two major uses of telemedicine: the first is to support patients, and the second is to support caregivers^20^. Telemedicine is used to make virtual recurring contact with ADRD patients and their caregivers at suitable intervals to check on progress made in the treatment since the last appointment with the health practitioner. Appropriate follow-ups in dementia care can help adjust treatments, maintain a good social relationship with the patient, and adjust treatments based on further tests^21^. Supporting patients has played a huge role in improving their cognitive and motor functions^20^. Additionally, the Resources for Enhancing Alzheimer's Caregiver Health (REACH) project was introduced to evaluate possible solutions for enhancing and providing adequate support for caregivers of ADRD patients^22^. We explored four papers related to the efficacy of the REACH project. One study revealed that participants who received the intervention experienced a decrease in burden and an increase in their appreciation of providing caregiving and social support for the patient^23^. Another study reported a decrease in depression and a substantial increase in self-efficacy among the participants in the program^24^. These findings are comparable to two other studies, the DE REACH by Berwig et al.^25^ and the REACH VA by Nichols et al.^26^. All the reports showed the importance of telecommunications devices, especially the telephone and video conferencing, in enhancing caregiving.

## ACCESSIBILITY OF TELEMEDICINE IN DEMENTIA TREATMENT AND CARE

Telemedicine is a rapidly expanding paradigm for the treatment of sick people, including victims of dementia with guardians^27^ and, most notably, for patient-care standards disrupted by the COVID-19 pandemic^28^. Clinical examinations — critical in the care of elderly with ADRD — adapt to telemedicine using synchronous videoconferencing, which allows doctors to see a dementia victim's home setting^29^ while keeping a safe distance to protect this vulnerable group^30^. The effectiveness of this type of treatment, on the other hand, is contingent on the patient's capacity to see, hear, and understand the physician, in addition to having reliable access to technology, as these are the required factors to connect with the clinician^31^. The criteria have caused various issues for the elderly with ADRD due to varying requirements when dealing with telemedicine networks as a result of age-related changes in mental ability^32^.

Additionally, hearing loss due to aging is frequent, with up to 90% of those with dementia suffering from it^33^. Age-related hearing loss, usually untreated, might make it more difficult for doctors to measure cognitive function^34,35^. Visual impairment is very common as well, affecting more than 30% of dementia patients. Globally, about 2.1 million people have moderate or severe vision impairment^36^, and 11.3% of people aged 80 years and above have double sensory damage, thus, increasing the risk of dementia^37^.

Furthermore, the success of telemedicine relies on the caregivers’ technology skills and the patients’ ability to communicate virtually with the doctor. The additional technological skills that telemedicine requires may hinder its use as a regular care therapy modality for elderly victims with dementia. As telemedicine becomes more widely used in the future, elderly victims could be immediately excluded if the appropriate adjustments to the technology and sensory requirements are not made for them. Taking into account this population's technical and sensory needs also opens doors to expand dementia care access and reduce the stress of money issues, logistics, and trip periods for individuals and guardians^38^. The high transmission rate of COVID-19 led to an increased demand for in-home telemedicine. This highlights the importance of ongoing research aimed at improving in-home telemedicine delivery and standardizing it as a component of medical treatment^28^. Previous research on telemedicine applications in dementia patients has primarily focused on reducing guardians’ workload and discomfort, with only short attention paid to the patients’ experience^27^. Future initiatives to enable older people with ADRD to participate in telemedicine may benefit from a closer examination of the victim's specific concerns about technology attainability and sensational requirements^38^. As people with dementia increasingly struggle to process and respond to impulses, and as pressure can be caused by alterations in activities and surroundings, studies involving telemedicine visits carried out at various residences found that apparatus arrangement, orientation, and testing before the technology usage assisted in adapting the users to the surroundings^39^.

The guardians could greatly help create an ideal dwelling environment for the telecare, by reducing disturbance and a noisy environment and streamlining activities for the victim. Prior to the meeting, orientation and training can help the care partner set clear expectations and lessen the novelty of the scenario for the dementia patient. To ensure that the evolution of novel performances and services on telemedicine platforms is user-driven, victims and guardians should offer insightful suggestions^38^. The strain of travel and out-of-home visits for victims and their guardians can be lessened in the near future by improving telemedicine. Recent research on the COVID-19 pandemics effects on physical distance, however, has emphasized the increasing difficulties for guardians and the cessation of routine mental and bodily therapy for patients^40^. According to another study, older people with dementia who experience prolonged physical isolation may present worsening neuropsychiatric symptoms^41^. Even though telemedicine has demonstrated numerous benefits in this current crisis, long-term solutions to telehealth provision challenges require equal attention given their potential to make dementia care more accessible to victims and their guardians (Table 1)^38,42–47^.

**Table 1:** The table below shows a summary of the benefits and challenges of telemedicine in dementia care.

Benefits of the application of telemedicine in dementia care.	Challenges encountered in the application of telemedicine in dementia care.
Telemedicine helps slow the cognitive decline in patients with dementia. This was shown in a study conducted by Kim et al., whereby they evaluated how effective telemedicine can be in relation to changes in the cognition of dementia patients^42^. Telemedicine has the capability of bridging the gap between dementia patients and their access to specialized support, which is being caused by the dearth of specialist support for dementia patients^43,44^. Telemedicine helps put a check on the rate of undiagnosed patients with dementia, which can be attributed to the shortage of geriatric psychiatrists^45^. Telemedicine, in that it serves as a means of healthcare delivery, also improves the general wellbeing of dementia patients as well as their caregivers^46^.	Lack of access to telecommunication technologies that will subsequently be used to harness the benefits of telemedicine^47^. The effect of dementia may be exacerbated by the patient's loss of hearing and sight, severely limiting how telemedicine can be used to provide patient care^38^. The mode of telemedicine delivery in most LMICs is not accessible to dementia patients in the region.

## CURRENT IMPROVEMENTS IN TELEMEDICINE IN THE MANAGEMENT OF DEMENTIA

Although telemedicine has been found to be a convenient and low-cost approach in dementia treatment^46,48^, it requires improvement in a variety of areas in order to maximize its use. The cost of accessing telemedicine through the use of telecommunications technologies should be made affordable. Most dementia patients in low- and middle-income countries are unable to afford telehealth services either because they are unaffordable or because they are unavailable due to poverty and subpar healthcare systems in these countries. In response to this, various governments should allocate funds to the telemedicine and e-health sections of the healthcare system in their countries, and subsidization should be provided for telehealth services and equipment to make them affordable.

Furthermore, in some parts of the world, such in West Africa, Alain reported in his published work that less than 5% of the households own a computer, while those that have access to a mobile network are only 41.5%^49^. Although there are few studies and statistical reports that show the exact percentage of dementia patients in West Africa, studies evidence that this disease is extremely common among citizens of low and middle-income countries (LMICs), of which West African countries are primarily part^1^. Even though telemedicine is being used to treat dementia, its potential cannot be fully performed due to the low percentage of the population that has the necessary telecommunication technologies. Furthermore, a significant barrier to the telemedicine accessibility for dementia patients is the poor internet connectivity in most urban regions and the restricted availability of broadband internet in the least developed nations (LDC). According to estimates, 27% of people in the LDCs had access to broadband internet in 2021, making them the region with the fewest connections^50^. Therefore, governments and stakeholders should make internet infrastructure resources available in these areas; additionally, the implementation of 5G networks will offer a long-term solution to the lack of internet connectivity in urban areas.

More so, the sense of companionship should be more integrated into the technological tools used in telemedicine. Blazer, in his work, identified “social isolation” and “loneliness” as factors that lead to a high risk of developing cognitive disorders^51^. In a similar vein, Goodman-Casanova et al. found that the isolation of dementia patients caused sleep disorders and negative feelings in their study conducted in Spain^52^. This can be improved by making health and social services available to dementia patients and building a strong support network around them, which can be done through social media, which is coincidentally being accessed with the same tools as telemedicine.

Moreover, infrastructures that will enable the use of telemedicine should be adequately deployed. Infrastructures that support the usage of telemedicine, like good internet access, network coverage, and an adequate supply of electricity, should be improved in order to effectively and efficiently employ telemedicine in dementia treatment. In his statistical report, published on “Sustainable Energy for All”, Puliti stated that West Africa is the region with the lowest rate of electricity access: “Only 42% of the total population and 8% of rural residents have access to electricity^53^.”

Telemedicine application in dementia treatment may be limited if end users are unaware of and unfamiliar with modern telecommunication technologies^54^. Therefore, for an improved application of telemedicine in dementia treatment, patients are required to have a good grasp of telecommunication technologies; but these may not be totally obtainable due to the loss of cognition, thus, caregivers can be of help in this aspect^55^.

In conclusion, telemedicine has been shown to have a significant impact on dementia treatment because it allows for timely and equitable treatment. Studies have also demonstrated its effectiveness in terms of patients’ diagnosis, follow-up, treatment, and caregiver support. Additionally, its accessibility was found to have some advantages in its use, since the majority of telemedicine applications are widely used in developed nations in Europe and the Americas. However, in the majority of African nations and some other less developed nations in other parts of the world, telemedicine is understudied and has few applications, primarily because of the subpar healthcare systems and highly inaccessible mode of its delivery in these nations. Therefore, there is a need to improve support, accessibility, education, and empowerment in order to remove obstacles and promote the adoption of e-health in the treatment of people with ADRD^11^. Finally, since this will boost telemedicine's acceptance and use, coordinated efforts should be made to present accurate research on the subject in understudied regions.
